# Sodium Caseinate and Acetylated Mung Bean Starch for the Encapsulation of Lutein: Enhanced Solubility and Stability of Lutein

**DOI:** 10.3390/foods11010065

**Published:** 2021-12-28

**Authors:** Yifan Lu, Bo Zhang, Huishan Shen, Xiangzhen Ge, Xiangxiang Sun, Qian Zhang, Xiuyun Zhang, Zhuangzhuang Sun, Wenhao Li

**Affiliations:** College of Food Science and Engineering, Northwest A&F University, Xianyang 712100, China; luyifan0222@163.com (Y.L.); zhangbo383@163.com (B.Z.); shen1685778117@163.com (H.S.); 18393810565@163.com (X.G.); sx1141151470@163.com (X.S.); z-grace@nwafu.edu.cn (Q.Z.); zhangxiuyun123415@163.com (X.Z.); Sunzhuangz@nwafu.edn.cn (Z.S.)

**Keywords:** lutein, sodium caseinate, acetylated mung bean starch, microcapsule

## Abstract

Lutein is a kind of vital carotenoid with high safety and significant advantages in biological functions. However, poor water solubility and stability of lutein have limited its application. This study selected different weight ratios of sodium caseinate to acetylated mung bean starch (10:0, 9:1, 7:3, 5:5, 3:7, 1:9, and 0:10) to prepare lutein emulsions, and the microcapsules were produced by spray drying technology. The microstructure, physicochemical properties, and storage stability of microcapsules were investigated. The results show that the emulsion systems were typical non-Newtonian fluids. Lutein microcapsules were light yellow fine powder with smooth and relatively complete particle surface. The increase of sodium caseinate content led to the enhanced emulsion effect of the emulsion and the yield and solubility of microcapsules increased, and wettability and the average particle size became smaller. The encapsulation efficiency of lutein microcapsules ranged from 69.72% to 89.44%. The thermal characteristics analysis showed that the endothermic transition of lutein microcapsules occurred at about 125 °C. The microcapsules with sodium caseinate as single wall material had the worst stability. Thus, it provides a reference for expanding the application of lutein in food, biological, pharmaceutical, and other industries and improving the stability and water dispersion of other lipid-soluble active ingredients.

## 1. Introduction

Lutein is a fat-soluble carotenoid, insoluble in water, and slightly soluble in some oils. Lutein widely exists in egg yolk, fruits, vegetables, flowers, and other plants [1]. The structure of lutein can be described as a long carbon chain containing 40 carbon atoms, with methyl side groups, alternating single carbon and double carbon bonds, and two hydroxyl groups connected at both ends of the long chain. Lutein has intense antioxidant activity and is conducive to improving the body’s own immune ability [2], slowing down the aging of cells and organs, and protecting the skin from the sun [3]. As a result, lutein can effectively prevent or relieve a range of human diseases, such as ulcers, strokes, breast cancer, lung cancer, atherosclerosis, heart disease, age-related macular disease, cataract, and night blindness. Lutein can also be used as a colorant because of its bright orange yellow. Lutein is usually used in food, feed, cosmetics, medicine, and other industries. However, lutein contains many double carbon bonds, resulting in poor stability and easy degradation, weakening or even losing its biological activity. The water insolubility of lutein also limits its application. Therefore, it is crucial to find appropriate methods to improve the stability and solubility of lutein to make better use of lutein and develop and design new functional foods.

Microencapsulation has been proven to be an excellent method to enhance the stability and solubility of labile bioactive components [4]. The composition and selection of wall materials are essential to the properties of microcapsules. Carbohydrates, hydrophilic colloids, and proteins are common wall materials. For lutein microencapsulation, wall materials from various sources have been tried. Zhu et al. [5] reported that β-carotene, lutein, zeaxanthin, and fish oil were co-encapsulated with whey protein isolate and octenylsuccinic anhydride modified starch as wall matrices. Ding et al. [6] reported that seven different carbohydrates, including sucrose, trehalose, inulin, three maltodextrins with different DE and modified starch, were used as lutein microencapsules wall material. Zhao et al. [7] prepared lutein-loaded sodium caseinate microcapsules and lecithin microcapsules. However, to our knowledge, no publications devoted to the encapsulation of lutein with sodium caseinate-acetylated mung bean starch complex as wall material exist.

Acetylated starch, a starch derivative produced by the reaction of starch and acetic anhydride in an alkaline environment, is a typical kind of esterified starch [8]. It has suitable film-forming properties, thermal stability, and emulsifying performance, expanding its application in the controlled-release system of active factors and microcapsule wall materials. It, therefore, has excellent potential as a microcapsule carrier. However, single wall material cannot fully meet the required performance of the product. Casein is the most abundant protein in milk. It is an unfolded irregular coiled protein [9]. Casein is a kind of ideal emulsifier because it arranges hydrophobic and hydrophilic amino acid residues [10]. Therefore, sodium caseinate is widely used in the delivery system, promoting emulsion formation and stabilizing it by reducing interfacial tension and forming a protective film around oil droplets [11]. In addition, sodium caseinate has outstanding functional properties, such as thickening, emulsifying, foaming, and thermal stability. However, sodium caseinate as a single wall material may not be able to meet the requirements for encapsulation of active substances because it is very sensitive to acidic pH close to its isoelectric point. This will have a negative impact on the stability of the emulsion. In order to overcome this limitation, researchers have adopted methods such as combining two materials as wall materials. Perugini et al. [12] found that the emulsions stabilized with sodium caseinate and Tween 20 were more stable, compared with emulsions stabilized only with sodium caseinate at pH close to caseinate isoelectric point. Cuomo et al. [13] found that the mixed emulsifier system offered a higher amount of lipophilic compound with a low fat intake compared to nanoemulsions stabilized by sodium caseinate.

Therefore, this study used sodium caseinate and acetylated mung bean starch as wall materials to prepare lutein microcapsules by spray drying. The morphological structure, physicochemical properties, and storage stability of microcapsules prepared by sodium caseinate and acetylated mung bean starch in different proportions (10:0, 9:1, 7:3, 5:5, 3:7, 1:9, and 0:10) were systematically compared and investigated. The results reported in this paper are useful to provide some potential parameters for the embedding of lutein and have particular guiding significance for the future research of lutein.

## 2. Materials and Methods

### 2.1. Materials

Mung bean was purchased from Ganzhou Kangrui Agricultural Products Co., Ltd. (Ganzhou, Jiangxi, China). Sodium caseinate (purity ≥ 95%) was obtained from Zhejiang Yinuo Biotechnology Co., Ltd. (Jinhua, Zhejiang, China). Nile red (purity ≥ 98%) was purchased from Shanghai Yuanye Biotechnology Co., Ltd. (Shanghai, China). In addition, Other chemicals were obtained from Yangling Sanli Reagent Co., Ltd. (Xianyang, Shaanxi, China).

### 2.2. Sample Preparation

#### 2.2.1. Mung Bean Starch Isolating

Starches from mung beans were isolated, according to Galvez et al. [14]. The mung beans that had been soaked in warm water were ground and filtered. After discarding the upper water layer, the filtrate was repeatedly added with water and precipitated several times until the upper layer was free of color. The settled starch was dried at 40 °C, and then was crushed and sieved (150 µm sieve). A sample of mung bean starch was collected.

#### 2.2.2. Preparation of Acetylated Starch

The method reported by Zamudio-Flores et al. [15] was modified and used to synthesize acetylated starch. First, the esterifying agent (12% of starch mass, a mixture of acetic anhydride, and glacial acetic acid (1:1)) was added dropwise into the starch emulsion (35% *w*/*w*) while maintaining pH between 8.0 and 8.5. Then 0.5 mol/L HCl was added to adjust the pH to 6.5. Acetylated starch was recovered by centrifugation at 3000× *g* at 25 °C for 10 min. The starch was washed with distilled water 3 times and with anhydrous ethanol 3 times. The material was dried at 40 °C, and crushed and sieved (150 µm sieve). The sample was stored in a vacuum bag.

#### 2.2.3. Preparation of Sodium Caseinate-Acetylated Mung Bean Starch–Lutein Emulsion

The sodium caseinate-acetylated mung bean starch–lutein emulsion was obtained by homogenization and ultrasonic treatment based on the results of preliminary experiments. Briefly, the aqueous phase and lipid phase were separately prepared. The aqueous phase consisted of sodium caseinate and acetylated mung bean starch, and its total mass was 396 g. The lipid phase included 40 mg of lutein (0.2% of wall materials mass) and 4 g of medium chain triglyceride. As emulsifiers, the concentration of sodium caseinate and acetylated mung bean starch in emulsion was 5% (*w*/*w*) and their weight ratio was set to be 10:0, 9:1, 7:3, 5:5, 3:7, 1:9, and 0:10, respectively. A certain amount of protein and distilled water were stirred in a water bath constant temperature magnetic stirrer (HCJ-2D, Changzhou Ruihua Instrument Manufacturing Co., Ltd., Changzhou, China) for 12 h to make it fully swell. A certain amount of modified starch and distilled water were heated in a water bath constant temperature magnetic stirrer at 70 °C for 30 min and then stirred at room temperature overnight. The lipid phase was melted at 180 °C and added to the aqueous phase drop by drop in 10 min after cooling while stirring them. The mixture was dispersed by a homogenizer (Ultra Turrax, IKA Staufen, Breisgau, Germany) at 10,000 rpm for 6 min. The temperature was controlled below 45 °C by an ice-water bath. Then it was sonicated at 460 W for 5 min to obtain sodium caseinate-acetylated mung bean starch–lutein emulsion.

#### 2.2.4. Preparation of Sodium Caseinate-Acetylated Mung Bean Starch–Lutein Microcapsules

**Figure 1:**
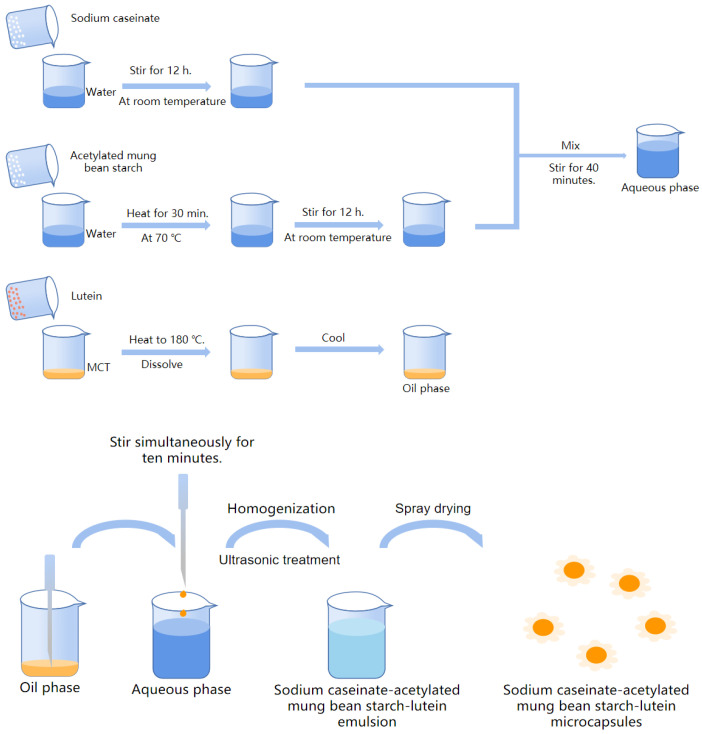
Schematics of lutein encapsulation using sodium caseinate and acetylated mung bean starch.

Figure 1 demonstrates how the lutein encapsulations were prepared. The emulsion prepared in part 2.2.3 was fed through a spray dryer (SP-1500, Shanghai Shunyi Experimental Equipment Co., Ltd., Shanghai, China). The operational conditions were as follows: inlet air temperature: 185 ± 5 °C, outlet air temperature: 85 ± 5 °C, feed rate: 400 mL/h [16]. Seven lutein microcapsules were labeled as SA10:0, SA9:1, SA7:3, SA5:5, SA3:7 and SA1:9 for sodium caseinate/acetylated mung bean starch at the weight ratio of 10:0, 9:1, 7:3, 5:5, 3:7 and 1:9. Emulsion prepared with sodium caseinate and acetylated mung bean starch at a weight ratio of 0:10 exhibited two phase separation after spray drying and failure to form microcapsules, and it was labeled E0:10. During the preparation process, the samples were wrapped with tin foil and carried out under the condition of avoiding light.

### 2.3. Characterization of Emulsion

#### 2.3.1. Optical Microscopy Observation for Emulsion

Emulsion morphology was observed by a digital optical microscope (DMBA400, Motic China Group Co., Ltd., Xiamen, China) at 400× magnification and took photos to record observations.

#### 2.3.2. Fluorescence Microscopy Observation for Emulsion

Fluorescence microscopy was carried out using an automatic fluorescence microscope (LECIA DM6 B, Lecia, Wetzlar, Germany). Nile red (1 mg) was dissolved in 1 mL of DMSO to prepare a fluorescent dye solution. The fleshly prepared emulsion and dye solution was mixed evenly and dropped on the slide, covered with a coverslip, and left to dry naturally for 2 h. The distribution of the two phases was observed using a fluorescence microscope.

#### 2.3.3. Rheological Property Determination

The rheological property of lutein emulsion was determined using a rotational rheometer (DHR-1, Waters, Milford, MA, USA). The model chosen was flow scanning. Each measurement was carried out using parallel plates with a 40-mm gap. The temperature was set at 25 °C and 50 °C, respectively. Dynamic viscosity values were obtained at shear rates in the range of 0.01–100 s^−1^. The flow measurement data were evaluated by Herschel–Bulkley:

(1) $$\tau \;={\;\tau }_{0}+{K\gamma }^{n}$$

where τ is the shear stress (Pa); τ_0_ is the yield stress (Pa); K is the consistency coefficient (Pa·s^n^); γ is the shear rate (s^−1^); n is the flow behavior index.

### 2.4. Characterization of Microcapsule

#### 2.4.1. Scanning Electron Microscopy (SEM) for Microcapsules

The morphology and inner structure of the prepared spray-dried microcapsule powders were determined with a scanning electron microscope (Nova Nano SEM 450, FEI, Hillsboro, OR, USA). The samples were immobilized on an aluminum stage with a conductive double-sided tape and metalized with a thin layer of gold [17].

#### 2.4.2. Fourier-Transform Infrared Spectroscopy (FTIR) for Microcapsules

The FT-IR analysis was carried out in a Fourier transform infrared spectrometer (VERTEX70, German BRUKER Company, Karlsruhe, Germany) with 4 cm^−1^ resolution at a spectral range of 4000–400 cm^−1^ using KBr plates [7].

#### 2.4.3. Determination of Yield

The yield of lutein microcapsules is the ratio of the final mass of microcapsules after spray drying to the initial mass of the sample before spray drying. The yield (Y) was calculated using Equation (2).

(2) $$Y\;\left(\%\right)=\frac{\mathrm{The}\;\mathrm{final}\;\mathrm{weight}\;\mathrm{of}\;\mathrm{the}\;\mathrm{microcapsules}\;\mathrm{after}\;\mathrm{spray}\;\mathrm{drying}\;(g)}{\mathrm{The}\;\mathrm{total}\;\mathrm{weight}\;\mathrm{of}\;\mathrm{the}\;\mathrm{sample}\;\mathrm{added}\;\mathrm{before}\;\mathrm{spray}\;\mathrm{drying}\;\left(g\right)}\times 100\;$$

#### 2.4.4. Determination of Encapsulation Efficiency

The encapsulation efficiency (EE) of lutein microcapsules was determined according to the method of Bejrapha et al. [18] and was slightly changed. Lutein was dissolved in absolute ethanol and formulated into solutions of different concentrations. The absorption of the solutions was measured using a dual-beam ultraviolet/visible spectrometer at 446 nm with ethanol solution (95%, *v*/*v*) as a blank. A plot of absorbance of lutein vs. concentration of lutein was established to make the standard curves, which was described by the equation A = 2.145C + 0.0011 (R^2^ = 1). Accurately weighed lutein microcapsule granules (20 mg) were solubilized in 1 mL of deionized water by ultrasound-assisted method and diluted with absolute ethanol to 20 mL. Lutein microcapsule granules (30 mg) were accurately weighed and diluted with absolute ethanol to 20 mL while shaking for 1 min. Then these two solutions were centrifuged (4000 rpm, 10 min) at 4 °C. The absorbance of the supernatant was determined to calculate the total lutein content and lutein content on the microcapsules’ surface. Encapsulation efficiency was calculated quantitatively using Equation (3):

(3) $$\mathrm{EE}\;\left(\%\right)=\left[1-\frac{\mathrm{surface}\;\mathrm{lutein}\;\mathrm{content}\;\mathrm{in}\;\mathrm{the}\;\mathrm{powder}}{\mathrm{total}\;\mathrm{lutein}\;\mathrm{content}\;\mathrm{in}\;\mathrm{the}\;\mathrm{powder}}\right]\times 100\;$$

#### 2.4.5. Measurement of Moisture Content

The moisture content of the dried microcapsules was determined using the vacuum drying oven (DZX-6053B, Shanghai Fuma Experimental Equipment Co., Ltd., Shanghai, China) at 105 °C until constant weight. The moisture content was determined following the official methods of the GB 5009.3-2016.

#### 2.4.6. Measurement of Solubility

The lutein microcapsules (dry basis) were solubilized in deionized water and were centrifuged (4000 rpm, 10 min) at 4 °C. Then the supernatant was poured out. After several repetitions, the residue was washed with deionized water into an aluminum box of known quality and dried to a constant weight. The solubility (S) was calculated using Equation (4).

(4) $$S\;\left(\%\right)=\left[1-\frac{\mathrm{The}\;\mathrm{sum}\;\mathrm{of}\;\mathrm{the}\;\mathrm{weight}\;\mathrm{of}\;\mathrm{the}\;\mathrm{aluminum}\;\mathrm{boxes}\;\mathrm{and}\;\mathrm{insoluble}-\mathrm{Weight}\;\mathrm{of}\;\mathrm{the}\;\mathrm{aluminum}\;\mathrm{boxes}}{\mathrm{Weight}\;\mathrm{of}\;\mathrm{the}\;\mathrm{samples}}\right]\times 100\;$$

#### 2.4.7. Measurement of Wettability

Accurately weighed microcapsule powders (0.1 g) were sprinkled in distilled water (50 mL) at room temperature and then stirred on a magnetic stirrer at 450 rpm until it was completely submerged. The time of complete immersion of the powders was recorded [19].

#### 2.4.8. Color Characterization

The color of the microcapsules was measured with a chromameter (Ci7600, Aiseli (Shanghai) color technology Co., Ltd., Shanghai, China). A 10 mm reflecting ring was selected as the measuring hole. The results were expressed as luminosity (L*), redness (+a*) or greenness (−a*), and yellowness (+b*) or blueness (−b*). The microcapsule SA10:0 was used as the standard sample. The total color difference ∆E was calculated according to the following Equation (5). All measurements were performed in triplicates.

(5) $$\Delta E=\sqrt{{\left(L^{*}-L_{0}^{*}\right)}^{2}+{\left(a^{*}-a_{0}^{*}\right)}^{2}+{\left(b^{*}-b_{0}^{*}\right)}^{2}}$$

where, L*, a* and b* represent the color of the microcapsule samples; L_0_*, a_0_* and b_0_* represent the color of microcapsule samples prepared with protein as single wall material.

#### 2.4.9. Particle Size Distribution

The particle size distribution of prepared lutein microcapsules was determined by a laser particle size analyzer (Coulter LS-13320, Beckman Coulter, Miami, FL, USA) after diluting with distilled water. The selected sample processing module was the Universal Liquid Module (ULM). These measurements were performed in triplicate.

#### 2.4.10. Differential Scanning Calorimetry (DSC) Analysis

Differential scanning calorimetry (DSC) was performed using a differential scanning calorimeter (Q2000, Waters, Milford, MA, USA). Accurately weighed lutein microcapsule samples (3 mg) were placed in a hermetic-type aluminum crucible. An empty, sealed crucible was used as a reference. Nitrogen atmosphere at a 20 mL/min flow and a heating rate of 10 °C/min was applied from 30 °C to 250 °C [20].

### 2.5. Stability Tests

#### 2.5.1. Influence of Temperature on the Retention Rate of Microcapsules

Three kinds of lutein samples with a high encapsulation efficiency were stored at 4 °C, 25 °C, and 50 °C for 15 days. 100 mg of microcapsule samples were collected on days 3, 6, 9, 12, and 15, respectively. After the thermal stability test, the total lutein content in the samples was determined by the method mentioned in part 2.4.2. The retention rate of lutein in microcapsules was calculated according to the following Equation (6).

(6) $$R\;\left(\%\right)=\frac{\mathrm{Residual}\;\mathrm{lutein}\;\mathrm{content}}{\mathrm{Initial}\;\mathrm{lutein}\;\mathrm{content}}\times 100$$

#### 2.5.2. Influence of Light on the Retention Rate of Microcapsules

The light stability test was performed using two light sources, namely an ultraviolet light lamp and a cold light fluorescent lamp installed in a chamber. Three kinds of microcapsules with a high encapsulation efficiency were exposed to constant brightness for 15 d. 100 mg of microcapsule samples were collected on days 3, 6, 9, 12, and 15, respectively. Quantification of the total lutein content was performed, and the retention rate of lutein in microcapsules was calculated as previously described.

### 2.6. Statistical Analysis

All experiments and determinations were performed in triplicate, and the results were reported as average ± standard deviation. Experimental data were subject to Tukey’s multiple range tests using Minitab version 18.1 package (Minitab Inc., State College, PA, USA), and the confidence level was set at 95%. The SPSS statistical software (version 20.0, SPSS Inc., Chicago, IL, USA) was used for performing the principal component analysis (PCA).

## 3. Results and Discussion

### 3.1. Characterization of Emulsion

#### 3.1.1. Optical Microscopy Observation for Emulsion

**Figure 2:**
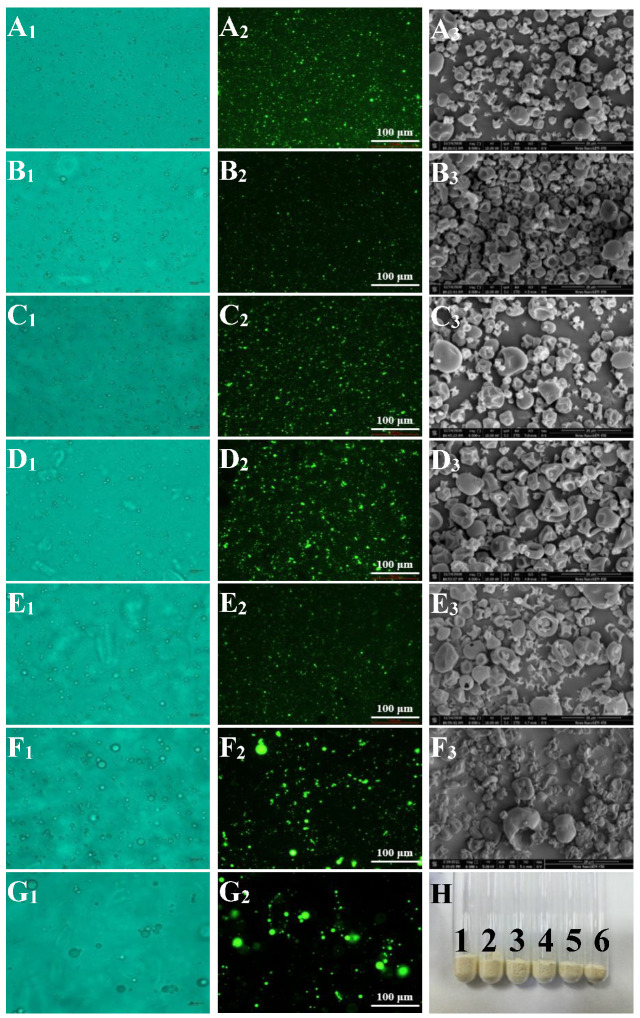
Light micrographs (×400) (1) and fluorescence micrographs (AFM) (×200) (2) of sodium caseinate-acetylated mung bean starch–lutein emulsion, scanning electron micrographs (SEM) (×6000) (3) and appearance (H) of microcapsules. The letters from A to G indicate that the weight ratios of sodium caseinate and acetylated mung bean starch are 10:0, 9:1, 7:3, 5:5, 3:7, 1:9, and 0:10, respectively. Number from 1 to 6 indicate that the weight ratios of sodium caseinate and acetylated mung bean starch are 10:0, 9:1, 7:3, 5:5, 3:7, and 1:9, respectively.

A_1_-G_1_ in Figure 2 shows the morphology of lutein emulsions prepared using sodium caseinate and acetylated mung bean starch as emulsifiers under the optical microscope. The emulsifier can be miscible in the oil and water phases, which helps form tiny and uniform emulsion droplets and form a colloidal system with stable motion [21]. Sodium caseinate and acetylated mung bean starch can reduce the interfacial tension, form a repulsive layer between droplets, prevent aggregation, and promote the decomposition of large droplets when adsorbing to the oil-water interface [22].

The droplets of emulsions with a high concentration of sodium caseinate were tiny and uniform, indicating that the emulsification effect was better and effectively prevented the fusion between droplets. The increased modified starch content in emulsions enabled large droplets to appear in the lutein emulsions prepared with sodium caseinate and acetylated mung bean at a weight ratio of 1:9 and 0:10. Compared with the other emulsions, larger droplets and aggregates appeared with acetylated mung bean starch as single wall material, indicating that the emulsification effect of the emulsions with more modified starch was poor. Perhaps this has to do with the lower interfacial strain between two phases in the emulsion with a high concentration of sodium caseinate. According to the Laplace theorem [23], its droplets are smaller under the condition of constant homogenization. Besides, the peptide branches covalently bind to the oil droplets and other compounds in the emulsion to form smaller particles [24]. It is speculated that there were more peptide branches in the emulsion with high sodium caseinate content.

#### 3.1.2. Fluorescence Microscopy Observation for Emulsion

A_2_-G_2_ in Figure 2 were the fluorescence micrographs of sodium caseinate-acetylated mung bean starch–lutein emulsions. The oil droplet aggregation phenomenon was evident in the emulsions prepared with sodium caseinate and acetylated mung bean starch at a weight ratio of 1:9 and 0:10. The oil droplets in other emulsion systems were smaller and evenly distributed. The results showed that the emulsifying effect of emulsions with sodium caseinate and a small amount of acetylated mung bean starch as emulsifiers was better. In comparison, the emulsifying effect of single acetylated mung bean starch as an emulsifier was poorer. These results were consistent with the results in part 3.1.1. and later part 3.4. However, in the study of Xu et al. [25], they prepared the lutein-enriched high internal phase emulsions stabilized by egg yolk-modified starch complex and observed the microstructure by a confocal laser scanning microscopy. They found the size of oil droplets decreased with the increase of the modified starch concentration and believed that this could be attributed to a greater number of modified starch molecules adsorbed onto the oil droplet interface, promoting an increased electrostatic and steric repulsion between the oil droplets. Further research is needed on the microstructure of protein-modified starch lutein emulsion.

#### 3.1.3. Rheological Properties

**Figure 3:**
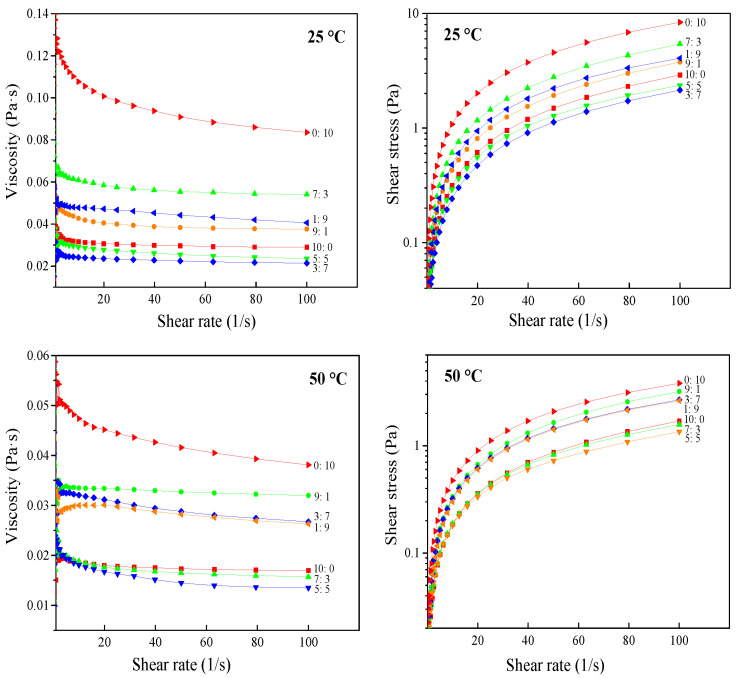
Shear rheological curve of sodium caseinate-acetylated mung bean starch–lutein emulsion. 10:0, 9:1, 7:3, 5:5, 3:7, 1:9, and 0:10, respectively, represent the weight ratio of sodium caseinate and acetylated mung bean starch.

The rheological properties of lutein emulsions prepared by sodium caseinate combined and acetylated mung bean starch as emulsifiers at 25 °C and 50 °C were presented in Figure 3. Herschel–Bulkley model was used to evaluate the flow measurement data, and flow parameters were shown in Table 1. This model described the flow behavior well with good fitment of data as revealed by high R^2^ values. For this model, the shear-thinning behavior will take place when the flow behavior index (*n*) is below 1, while the shear thickening will occur when *n* > 1. A fluid with (*n* = 1) results in a Newtonian behavior with an initial yield value [26]. The rheological curves protruded to the shear stress axis to varying degrees, and all emulsions’ ‘n’ values were less than 1 (Table 1). In addition, the viscosity decreased with the increase of shear rate, indicating that the lutein emulsions exhibited a non-Newtonian shear thinning behavior. Shear induced destruction of emulsion structure, resulting in shear thinning region. This is related to the mechanism of oil drop deflocculation [27]. In the study of Batista et al. [28], emulsions containing various concentrations of lutein were prepared with vegetable oil, deionized water, pea protein isolate, and lutein. The emulsions’ steady-state flow curves presented a shear-thinning behavior with a zero-shear rate limiting-viscosity at very low shear rates.

The ‘K’ value of the emulsions prepared with acetylated mung bean starch as a single emulsifier was the largest, indicating that a strong and dense three-dimensional network structure had been formed, and the thickening effect increased. The ‘K’ value of lutein emulsions at 25 °C was higher than that at 50 °C, and most emulsions’ ‘*n*’ value was just the opposite. It demonstrated that temperature could affect the consistency and fluidity of emulsions. At lower temperatures, the strong linking effect of protein and starch kept the emulsions stable, and the degree of tensile deformation was lower under stress. In this case, lutein emulsion had a higher ‘K’ value and lower ‘*n*’ value.

### 3.2. Characterization of Microcapsules

#### 3.2.1. Scanning Electron Microscopy (SEM) Images for Microcapsules

SEM examined the surface morphology and microstructure of the lutein microcapsule. Microcapsules SA0:10 exhibited two phase separation after spray drying and failure to form microcapsules. Some physicochemical properties of microcapsules are related to the morphology of microcapsule powders. Therefore, it is necessary to analyze the morphology of lutein microcapsule powders. The color and dispersion of lutein microcapsules were uniform, and the powders were fine and fluffy without agglomeration (A_3_–F_3_ in Figure 2). The microcapsule particles were nearly spherical, and the membrane structure on the surface was dense and continuous. Nevertheless, their particle sizes were uneven. Some microcapsule particles had a concavity, and wrinkled surface, which could be due to rapid evaporation of moisture during the drying process [29]. During this process, the contact area between the emulsion and the hot air was significantly increased by atomization.

The surface of most microencapsulated particles was smooth and relatively complete, without cracks, voids, and holes basically, indicating that microencapsulation was conducive to the retention of lutein. This finding agreed with Ding et al. [6], where the lutein microencapsulated powders showed a nearly spherical shape with some degree of concavities and good structural integrity, with no visible cracks or breakages on the outer surfaces. Similar results were also reported by Qv et al. [30] and Kuang et al. [16].

However, in the study of Ding et al., the microencapsulated powders containing modified starch exhibited more prominent collapses or more shrinkage [6]. They believe that the modified starch has higher molecular weight and stronger film-forming performance than sucrose, trehalose, inulin and maltodextrin, resulting in more shrinkage and larger collapse [7]. In this study, holes and damages appeared on the surface of some SA5:5, SA3:7, and SA1:9 microcapsules. This might be that the water evaporation rate of the modified starch in the wall material was faster, and a chapped phenomenon occurred while forming a dense membrane structure, resulting in broken pores on the particle surface. This enhanced the permeability of microcapsules, and caused a weakening of the degree of microencapsulation so that the content of lutein on the surface and the oxidative degradation rate of microcapsules increased.

#### 3.2.2. Fourier-Transform Infrared Spectroscopy (FTIR) for Microcapsules

**Figure 4:**
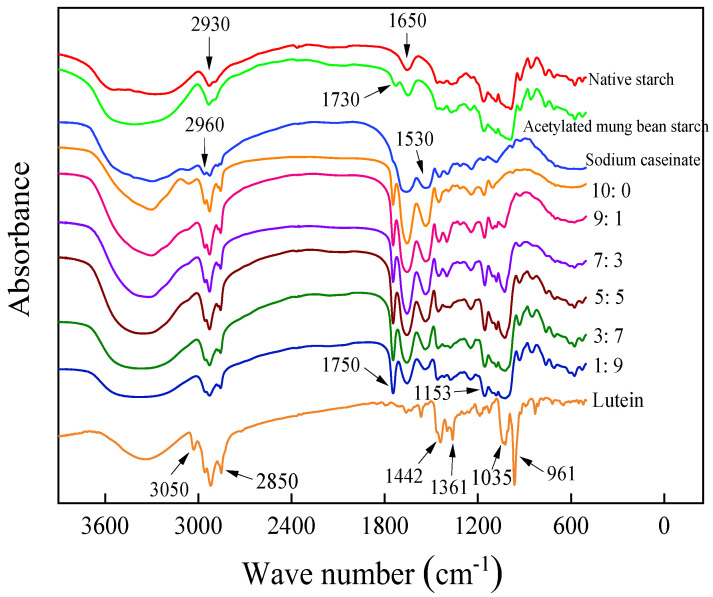
FTIR spectra of sodium caseinate-acetylated mung bean starch–lutein microcapsules. 10:0, 9:1, 7:3, 5:5, 3:7, and 1:9, respectively, represent the weight ratio of sodium caseinate and acetylated mung bean starch.

Experimental data with obtained spectra were presented in Figure 4. The spectra of natural mung bean starch and acetylated mung bean starch showed some similarities. The O-H stretching vibration attributed to hydrogen bonds was verified in the natural mung bean starch spectrum, between 3200 and 3600 cm^−1^. The peak at 2930 cm^−1^ and 1650 cm^−1^ corresponded to the CH symmetrical stretching vibrations and water bound in starch, respectively. Acetylated starch showed a peak representing C=O stretching of aliphatic esters at 1730 cm^−1^. It was proven that new groups were introduced into starch through esterification, and it also showed that the esterification reaction between raw starch and acetic acid occurred under alkaline conditions. The three peaks at 2960 cm^−1^, 2930 cm^−1^, and 2850 cm^−1^, indicating the =CH- stretching vibration, and two characteristic peaks at 1650 cm^−1^ and 1530 cm^−1^ were observed in the sodium caseinate spectrum.

In addition, four small peaks at 3050 cm^−1^, 2960 cm^−1^, 2930 cm^−1^, and 2850 cm^−1^, two splitting peaks at 1442 cm^−1^ and 1361 cm^−1^ representing the C–H stretching vibrations of CH_2_, and two peaks with high intensity at 1035 cm^−1^ and 961 cm^−1^ were all the characteristic absorption peaks of pure lutein. The obtained results agreed with those reported by Zhao et al. [7], who perceived a spectrum from the native sodium caseinate and lutein crystals very similar to the results of this study. In their research, the strong transmittance of lutein and sodium caseinate was at 1620.15 cm^−1^ and 1618.23 cm^−1^, respectively.

In addition to the characteristic peaks of wall materials, a new absorption peak appeared near 1750 cm^−1^ in lutein microcapsules (Figure 4). This absorption peak with high intensity and narrow shape was generated because of C=O stretching vibration. It was related to the embedding of lutein in the microcapsules, which proved that the lutein active component of the core materials had a strong binding effect with the wall materials to form a stable microcapsule system. Besides, there was an absorption peak with higher intensity and narrower shape than sodium caseinate at 2960 cm^−1^, 2930 cm^−1^, and 2850 cm^−1^ in the spectra of microcapsule powders. The spectrum of microcapsules exhibited C=O stretching at 1153 cm^−1^, which was related to the encapsulated lutein. The absent peak in the microcapsule FT-IR spectra at 961 cm^−1^, one of the characteristic peaks of pure lutein, was related to evidence that the lutein was deeply located within the microcapsule by binding with wall materials so that it cannot be detected. Similar results have also been described by Zhao et al. [7]. In their study, none of most bands of lutein were observed in the spectra of the lutein-loaded sodium caseinate microcapsules.

#### 3.2.3. The Yield, Encapsulation Efficiency, Moisture Content, Solubility and Wettability of Microcapsules

The yields of lutein microcapsules obtained in this study were between 22.49% and 79.64% (Table 2). The microcapsules prepared with sodium caseinate as single wall material had the highest yield. The yield of microcapsules decreased significantly with the increase of modified starch content (*p* ≤ 0.05). The yield can directly reflect the efficiency of spray drying. The products with low yield will stick to the drying chamber and increase the cost of cleaning, which harms production. This corresponded to the moisture content described later. The wall materials that are not easy to dehydrate will improve the viscosity of the materials and reduce the yield of microcapsules. Microcapsule SA1:9 had the highest moisture content, but its yield was the lowest. In the study of Ding et al. [6], the product yields of lutein microcapsule powders were between 55.6% and 92.6%. Microcapsules prepared with modified starch showed the highest yield after spray drying, while the microcapsule powders by sucrose, trehalose, and inulin gave the relatively lower product yield.

Encapsulation efficiency represents the ability of wall materials to enclose core components. Determining the packaging efficiency is conducive to study the application methods of effective packaging active materials [31]. Table 2 shows the EE of lutein in microcapsules. The encapsulation efficiency of lutein microcapsules ranged from 69.72% to 89.44%, reaching a high level, which indicated that the system had an excellent loading effect. The encapsulation efficiency of microcapsule SA7:3 was the highest, and microcapsule SA3:7 was the lowest. Similar findings were previously reported by Ding et al. [6] on lutein microencapsulated powders prepared with seven carbohydrates as an encapsulant. In their study, the lutein microencapsulated powders containing sucrose provided the lowest encapsulation efficiency because the reconstitution of sucrose crystal only involves one chemical constituent and lacks the capacity of embedding and protecting other chemical components. The lutein microencapsulated powders containing trehalose showed decent encapsulation efficiency due to its amorphous state after spray drying. For maltodextrin, lower DE may mean better encapsulation capacity. Kuang et al. [16] reported that EE varied from 69.1% to 90.1% among the lutein microcapsules encapsulated with maltodextrin and sucrose. The type and proportion of microcapsule wall materials will affect the embedding efficiency.

Moisture content is a critical parameter that plays a significant role in assessing powder quality, shelf life, and retention of bioactive compounds. Except for the moisture content of microcapsule SA1:9 being 4.45%, the moisture content of the other five microcapsule products was lower than 2.00%, reaching a deficient level, advantageous to the prevention against mildewing, moisture absorption, and accumulation. The difference in moisture content may be caused by the different affinity of materials for water or related to the different diffusion coefficients of water through the wall material. The high moisture content of microcapsules may be that the modified starch forms a shell structure in the fast-drying rate, making it difficult for the internal water to diffuse.

Good solubility is an essential factor in the application of microcapsule powders in food. From Table 2, the lutein microcapsules had high solubility, ranging from 74.59% to 97.31%. The solubility of the microcapsules prepared with sodium caseinate as single wall material was the highest among all the samples, which was 97.31%. This was consistent with the following particle size distribution results. The microcapsules with high sodium caseinate content had a smaller average particle size and greater solubility. As the content of acetylated mung bean starch increased, the solubility of the microcapsules decreased significantly (*p* ≤ 0.05). The mung bean starch modified by acetic anhydride and glacial acetic acid contains hydrophobic bonds, resulting in a decrease in the solubility of the microcapsule system. Pure lutein has been challenging to be used in the food industry because it is a fat-dissolved pigment and insoluble in water. The microencapsulated lutein has shown better water-soluble dispersibility, so that it could expand the application of lutein. The solubility of pure lutein and its microcapsules was similar to that reported by Wang et al. [32]. They reported that free lutein could not be dissolved with water as solvent at room temperature, while encapsulated lutein using a wall system consisting of gelatin and porous starch could be dissolved immediately after 120 s.

Wettability indicates the ability of the powder to interact with water molecules. Chew et al. [33] reported that the shorter the time for the powder to dissolute into the water, the better for its physical attribute in food processing. Table 2 showed that the wettability of microcapsule powders with high sodium caseinate content was poor. The microcapsules prepared with sodium caseinate as single wall material took the longest to dissolve in water, 328.65 s. Casein molecule is a mixture of monomer and complex, which is easy to aggregate in the aqueous dispersion of sodium caseinate. These monomers cannot thoroughly remove the hydrophobic surface when interacting with water [34]. Therefore, sodium caseinate is not easy to dissolve into the water at room temperature, limiting the dissolution of lutein microcapsules and prolonging complete dissolution in water. Due to the increase of modified starch content, the dissolution time of lutein powders decreased significantly, and the wettability increased significantly (*p* ≤ 0.05).

#### 3.2.4. Color Characterization

The color determination results of lutein microcapsule powders are shown in Table 3. The color of the microcapsules was described in terms of L*, a*, b*, and ΔE values. Lutein microcapsules are generally light yellow fine powder. The L* value of microcapsules SA10:0 and SA7:3 were 94.87 and 94.83, respectively, significantly higher than others (*p* ≤ 0.05). In other words, these two kinds of microcapsules had a higher luminance. The higher the luminance of the sample was, the less content of lutein exposed on the surface of the microcapsule was, indicating that the embedding effect was better, which was consistent with the results of encapsulation efficiency above.

a* value represents the redness and greenness of a sample and becomes higher when the sample is redder. The value of b* is a measure of the yellowness and blueness of a sample and becomes higher when the sample is yellower [35]. a* value and b* value of microcapsule SA1:9 was the largest, −0.75 and 36.58 respectively. It indicated that microcapsules SA1:9 tended to be redder and yellower when compared with the other five kinds of microcapsule products. Except for microcapsules SA9:1 and SA3:7, the value of ΔE increased with the increase in the level of modified starch, indicating that the color of microcapsules gradually approached dark yellow. There were also significant differences among all the microcapsules’ ΔE values except for SA9:1 and SA3:7 (*p* ≤ 0.05).

#### 3.2.5. Particle Size Distribution

The average particle size and particle size distribution of microspheres play a crucial role in the possible application of microcapsules in the food industry [36]. The particle size data for spray-dried lutein microcapsule powders are shown in Table 4. Mean particle diameters can represent the degree of coarse particle, and the particle size distribution can reflect the concentration and unevenness of powder grains. In addition, the more regular the morphology of particulate matter is, the closer the measured volume mean diameter (d_4,3_) and surface mean diameters (d_3,2_) are, and the more concentrated the particle size distribution is.

As seen from Table 4, the sizes of the microcapsule powders were at the microscale. During the preparation of lutein emulsions, high-speed shearing, ultrasonic, cavitation, and impact together enhance the interaction between protein and starch, breaking the interface between the core material and wall material, which promotes the combination of three components in the systems. In the microcapsule systems, the average particle size of microcapsules with high sodium caseinate content was smaller. The particle sizes of microcapsule SA3:7 and SA1:9 were significantly larger than that of other samples (*p* ≤ 0.05). These findings agreed with Ding et al. [6], where the lutein microencapsulated powders containing modified starch exhibited a relatively large particle size, which could be attributed to the somewhat higher viscosity of the embedding medium generated by modified starch.

The viscosity of the emulsion has been considered an essential influence factor on the particle size of the spray-dried powder. The higher emulsion viscosity leads to the formation of larger droplets during spray atomization and consequently forms larger particles during the drying process [19]. The emulsification and film-forming ability of wall materials will also lead to the ballooning of microcapsules during drying to increase the particle size by increasing internal air content [37]. Generally, the particle size of microcapsules is inhomogeneous and shows a wide range of distribution. As can be seen from Table 4, the particle size of lutein microcapsules was widely distributed in the range of 1–70 µm. Mean particle diameters (d_4,3_ and d_3,2_) in microcapsules SA3:7 and SA1:9 differed considerably, indicating the microcapsules had a wide range of particle size distribution and irregular surface morphologies.

#### 3.2.6. Thermal Characteristics Analysis

**Figure 5:**
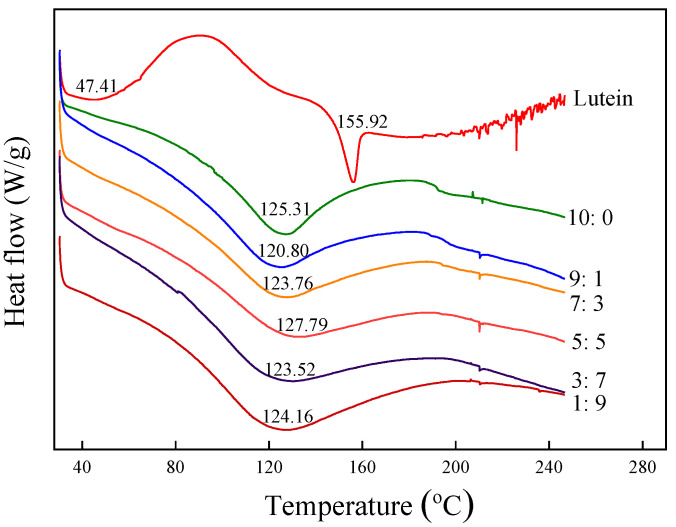
DSC thermograms of sodium caseinate-acetylated mung bean starch–lutein microcapsules. 10:0, 9:1, 7:3, 5:5, 3:7, and 1:9, respectively, represent the weight ratio of sodium caseinate and acetylated mung bean starch.

The DSC curves and glass transition temperature of microcapsules and pure lutein powders are shown in Figure 5. DSC thermogram of pure lutein powders demonstrated a quite obvious turning point starting phase change with Tg (peak temperature) at 47.41 °C. The DSC curve of lutein exhibited a sharp peak at 155.92 °C, corresponding to the melting point of lutein crystals. These results were similar to a study by Qv et al. [18] on the Tg (40.52 °C) of lutein product and an investigation by Zhao et al. [7] on the melting point (159.1 °C) of lutein crystals. The endothermic transition of lutein microcapsules occurred at about 125 °C, higher than Tg of pure lutein. The vitrification temperature assayed was higher than room temperature, which indicated that lutein microcapsules could maintain a relatively stable vitrification state under general storage conditions. It also revealed that lutein microcapsules could still maintain a complete structure and were not easily damaged during heat processing. This might be related to stable protein-modified starch–lutein complex formation. Tg of microcapsules SA5:5 was the highest in the six microcapsules, which was 127.79 °C. It demonstrated that the SA5:5 microcapsules needed the most heat for phase transition. That is, microcapsules SA5:5 had the most excellent thermal stability.

#### 3.2.7. Stability Tests

**Figure 6:**
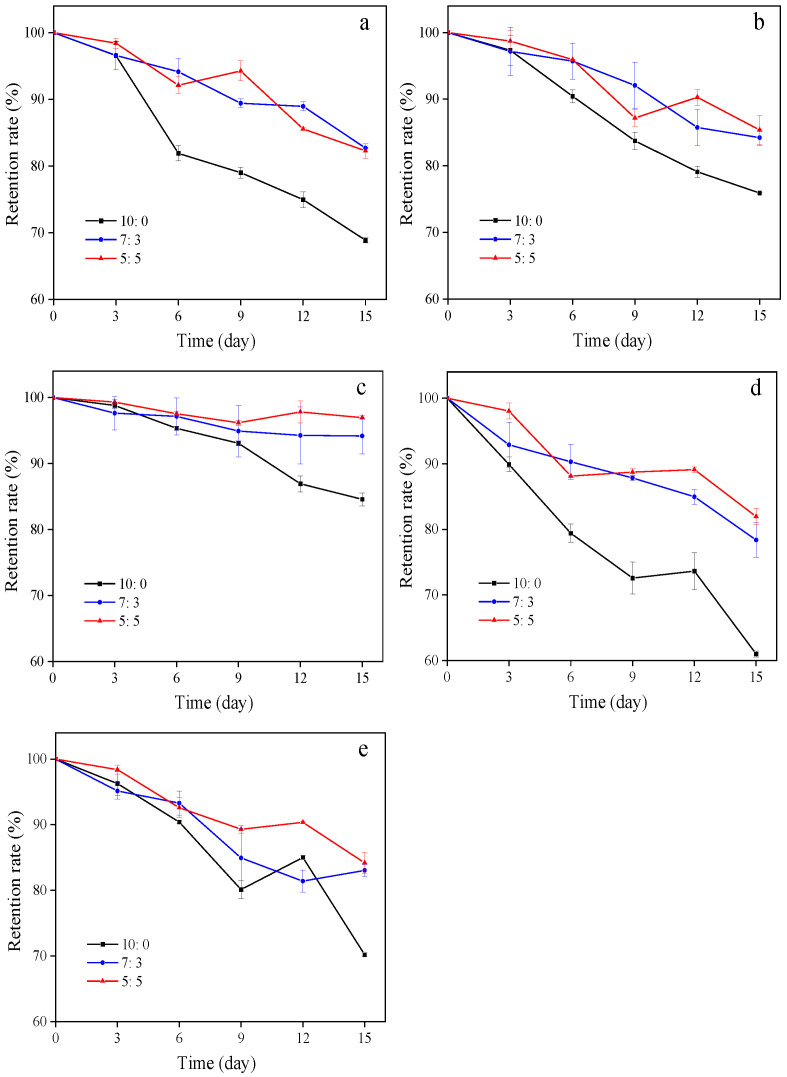
Retention rate of sodium caseinate-acetylated mung bean starch–lutein microcapsules under different storage conditions ((**a**), 50 °C in the dark; (**b**), 25 °C in the dark; (**c**), 4 °C in the dark; (**d**), UV light at room temperature; (**e**), lightness at room temperature). 10:0, 7:3, and 5:5, respectively, represent the weight ratio of sodium caseinate and acetylated mung bean starch.

Microcapsules with good stability have more application opportunities in industrial processing. Therefore, stability is an important index to evaluate the quality of microcapsules. Based on the actual conditions in processing, the preservation of lutein microcapsules in high temperatures, and the light environment was simulated. Figure 6 shows the stability data of three kinds of lutein microcapsules with a high loading rate stored at 4 °C, 25 °C, and 50 °C and under constant UV light and fluorescent lamp conditions for 15 d.

The temperature had remarkable effects on those heat-sensitive bioactive substances. The retention rate of lutein microcapsules decreases with the rise of temperature. The retention rates of microcapsules SA10:0, SA7:3, and SA5:5 stored at 50 °C for 15 days were 68.88%, 82.70%, and 82.29%, respectively. At 25 °C, the retention rates of microcapsules were 75.91%, 84.23%, and 85.38%, respectively. And they were 84.57%, 94.18%, and 96.96% at 4 °C. This indicated that temperature had a severe influence on the retention of lutein microcapsules. The microcapsules with sodium caseinate as single wall material had the worst thermal stability. Studies have shown that spray drying powders with smaller particle size and larger surface area showed faster degradation kinetics because the dissolution rate of active components embedded in the larger exposed area was higher [38,39]. The retention rate of SA10:0 at different temperatures was lower than that of SA7:3 and SA5:5. The possible reason was that SA10:0 had a smaller particle size and larger surface area, which increased the possibility of degradation and oxidation [5].

The retention rates of lutein microcapsules SA10:0, SA7:3, and SA5:5 were 61.01%, 78.38%, and 81.97%, respectively, when preserved under constant UV light conditions for 15 d, and were 70.18%, 83.04%, and 84.19%, respectively, when stored with a fluorescent lamp for 15 d. This shows the light exerts destructive influences on lutein. The light stability of microcapsules prepared with single sodium caseinate as wall materials was the worse, indicating that single wall material had limited ability to protect lutein from high temperature and being degraded by photooxidation.

Wang et al. [32] prepared lutein microcapsules with the porous starch and gelatin as wall materials, and they found that the lutein retention rate of lutein microcapsules had significantly been improved by about 46.9% and 32.2% than that of free lutein when studying the effect of heating time and illumination on lutein stability, respectively. This also shows that lutein microcapsules have better heat and light resistance stability than free lutein.

#### 3.2.8. Principal Component Analysis

**Figure 7:**
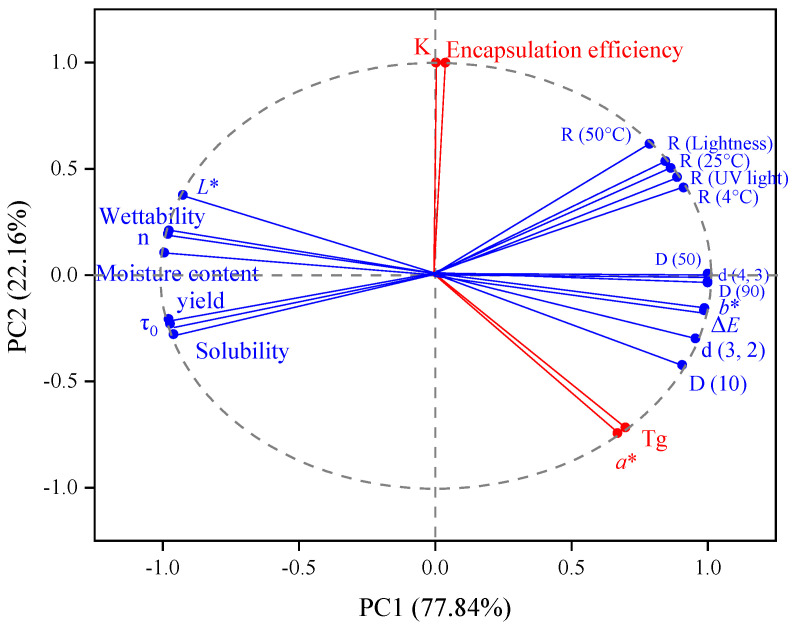
Principal component analysis of group differences.

The differences among the components of lutein microcapsules were contrasted by principal component analysis (PCA). As shown in Figure 7, no variables were directly related to the principal component. The contribution rate of the first principal component (PC1) was 77.84%, which mainly reflected the yield, moisture content, solubility, wettability, L*, b*, particle size distribution, flow behavior index (*n*), yield stress (τ_0_), and stability. The contribution rate of the second principal component (PC2) was 22.16%, which mainly reflected the encapsulation efficiency, glass transition temperature, consistency coefficient K, and a*. Thus, it showed that the components were interrelated and jointly affected microcapsules’ physical and chemical properties, consistent with the results above.

## 4. Conclusions

In this study, in order to overcome the defect of single wall material, different proportions of sodium caseinate and acetylated mung bean starch were selected to prepare lutein microcapsules. The properties of emulsion and microcapsule were studied. The results showed that with the increase of sodium caseinate content, the emulsifying effect of the emulsion increased, and the yield and solubility of the microcapsules increased. The highest encapsulation efficiency of microcapsules was 89.44%. The thermal characteristics analysis showed that the endothermic transition of lutein microcapsules occurred at about 125 °C, indicating that lutein microcapsules could maintain a relatively stable vitrification state under general storage conditions. Compared with the microcapsule prepared with sodium caseinate as single wall material, the microcapsules with sodium caseinate and acetylated mung bean starch as wall material were more stable. This study expanded the selection of lutein microcapsule wall materials and promoted the study of sodium caseinate in emulsion stability. The results from this study should be useful to design an ideal carrier to deliver lutein products with protein and polysaccharide as wall materials.

## Figures and Tables

**Table 1:** Fitting parameters of Herschel–Bulkley equation for sodium caseinate-acetylated mung bean starch–lutein emulsion.

Temperatures	Sample	τ_0_^1^ (Pa)	K ^2^ (Pa·s^−1^)	N ^3^	R^2,4^
25 °C	10:0	0.0055 ± 0.0001 ^a^	0.0333 ± 0.0000 ^f^	0.9695 ± 0.0001 ^a^	1.0000
	9:1	0.0029 ± 0.0009 ^ab^	0.0461 ± 0.0002 ^d^	0.9546 ± 0.0011 ^b^	0.9999
	7:3	0.0016 ± 0.0001 ^abc^	0.0667 ± 0.0000 ^b^	0.9539 ± 0.0001 ^b^	1.0000
	5:5	−0.0010 ± 0.0004 ^bc^	0.0376 ± 0.0000 ^e^	0.8992 ± 0.0003 ^e^	0.9999
	3:7	−0.0020 ± 0.0001 ^cd^	0.0287 ± 0.0001 ^g^	0.9369 ± 0.0005 ^c^	0.9999
	1:9	−0.0059 ± 0.0024 ^de^	0.0625 ± 0.0006 ^c^	0.9087 ± 0.0019 ^d^	0.9997
	0:10	−0.0083 ± 0.0013 ^e^	0.1437 ± 0.0005 ^a^	0.8829 ± 0.0008 ^f^	1.0000
50 °C	10:0	0.0026 ± 0.0007 ^ab^	0.0196 ± 0.0001 ^g^	0.9682 ± 0.0014 ^b^	0.9999
	9:1	−0.0014 ± 0.0004 ^c^	0.0363 ± 0.0001 ^d^	0.9731 ± 0.0004 ^a^	1.0000
	7:3	0.0010 ± 0.0006 ^b^	0.0218 ± 0.0001 ^f^	0.9284 ± 0.0012 ^c^	0.9999
	5:5	0.0032 ± 0.0003 ^a^	0.0236 ± 0.0001 ^e^	0.8762 ± 0.0006 ^g^	0.9996
	3:7	−0.0071 ± 0.0004 ^de^	0.0418 ± 0.0000 ^b^	0.9045 ± 0.0003 ^e^	0.9999
	1:9	−0.0086 ± 0.0000 ^e^	0.0389 ± 0.0000 ^c^	0.9172 ± 0.0000 ^d^	0.9998
	0:10	−0.0055 ± 0.0003 ^d^	0.0627 ± 0.0000 ^a^	0.8935 ± 0.0000 ^f^	0.9999

^1^ τ_0_ is the yield stress. ^2^ K is the consistency coefficient. ^3^ *n* is the flow behavior index. ^4^ R^2^ is the degree of fit. All the values are mean ± standard deviation (*n* = 3). Different letters at the same temperature in the same column indicate significant statistical differences (*p* < 0.05). 10:0, 9:1, 7:3, 5:5, 3:7, 1:9, and 0:10, respectively, represent the weight ratio of sodium caseinate and acetylated mung bean starch.

**Table 2:** The yield, encapsulation efficiency, moisture content, solubility, and wettability of sodium caseinate-acetylated mung bean starch–lutein microcapsules.

Sample	Yield (%)	EE ^1^ (%)	Moisture (%)	Solubility (%)	Wettability (s)
SA10:0	79.64 ± 0.84 ^a^	79.84 ± 0.20 ^b^	1.65 ± 0.01 ^b^	97.31 ± 0.00 ^a^	328.65 ± 8.73 ^a^
SA9:1	70.02 ± 0.29 ^b^	72.40 ± 0.49 ^c^	1.40 ± 0.27 ^bc^	94.98 ± 0.62 ^ab^	302.93 ± 4.84 ^b^
SA7:3	68.91 ± 0.25 ^c^	89.44 ± 0.81 ^a^	1.30 ± 0.08 ^bc^	93.92 ± 1.31 ^ab^	319.50 ± 4.69 ^ab^
SA5:5	61.13 ± 1.23 ^d^	81.41 ± 1.11 ^b^	0.50 ± 0.07 ^cd^	92.05 ± 2.61 ^b^	284.23 ± 5.66 ^c^
SA3:7	36.40 ± 1.39 ^e^	69.72 ± 2.83 ^c^	0.07 ± 0.00 ^d^	78.00 ± 0.02 ^c^	294.65 ± 4.51 ^c^
SA1:9	22.49 ± 0.57 ^f^	71.42 ± 1.32 ^c^	4.45 ± 0.58 ^a^	74.59 ± 0.34 ^c^	299.88 ± 0.14 ^bc^

^1^ EE is encapsulation efficiency. All the values are mean ± standard deviation (*n* = 3). Different letters in the same column indicate significant statistical differences (*p* < 0.05). SA10:0, SA9:1, SA7:3, SA5:5, SA3:7, and SA1:9 represent microcapsules with the weight ratio of sodium caseinate to acetylated mung bean starch of 10:0, 9:1, 7:3, 5:5, 3:7, and 1:9, respectively.

**Table 3:** The color of sodium caseinate-acetylated mung bean starch–lutein microcapsules.

Sample	L*	a*	b*	ΔE
SA10:0	94.87 ± 0.03 ^a^	−1.66 ± 0.05 ^cd^	18.59 ± 0.52 ^e^	0.60 ± 0.36 ^e^
SA9:1	93.22 ± 0.13 ^bc^	−1.24 ± 0.16 ^b^	30.74 ± 0.47 ^c^	12.74 ± 0.48 ^c^
SA7:3	94.83 ± 0.14 ^a^	−1.79 ± 0.04 ^d^	22.17 ± 0.33 ^d^	4.04 ± 0.33 ^d^
SA5:5	93.17 ± 0.09 ^c^	−1.48 ± 0.08 ^bc^	32.43 ± 0.35 ^b^	14.42 ± 0.36 ^b^
SA3:7	93.93 ± 0.06 ^b^	−1.71 ± 0.03 ^cd^	30.81 ± 0.35 ^c^	12.72 ± 0.35 ^c^
SA1:9	92.81 ± 0.61 ^c^	−0.75 ± 0.13 ^a^	36.58 ± 0.37 ^a^	18.50 ± 0.26 ^a^

All the values are mean ± standard deviation (*n* = 3). Different letters in the same column indicate significant statistical differences (*p* < 0.05). SA10:0, SA9:1, SA7:3, SA5:5, SA3:7, and SA1:9 represent microcapsules with the weight ratio of sodium caseinate to acetylated mung bean starch of 10:0, 9:1, 7:3, 5:5, 3:7, and 1:9, respectively.

**Table 4:** The mean particle size and size distribution of sodium caseinate-acetylated mung bean starch–lutein microcapsules.

Sample	d_(4,3)_ (μm)	d_(3,2)_ (μm)	Particle Size Distributions (μm)		
			D_(10)_	D_(50)_	D_(90)_
SA10:0	1.05 ± 0.01 ^c^	0.74 ± 0.01 ^d^	0.40 ± 0.01 ^c^	0.86 ± 0.00 ^c^	1.95 ± 0.00 ^d^
SA9:1	1.17 ± 0.07 ^c^	0.78 ± 0.01 ^d^	0.39 ± 0.01 ^c^	1.08 ± 0.09 ^c^	2.12 ± 0.12 ^d^
SA7:3	1.76 ± 0.23 ^c^	0.88 ± 0.14 ^d^	0.39 ± 0.07 ^c^	1.65 ± 0.18 ^c^	3.28 ± 0.48 ^cd^
SA5:5	2.96 ± 0.06 ^c^	1.96 ± 0.77 ^c^	0.69 ± 0.01 ^c^	2.81 ± 0.02 ^c^	5.56 ± 0.36 ^c^
SA3:7	23.00 ± 1.56 ^b^	6.37 ± 0.06 ^b^	4.52 ± 0.35 ^b^	23.47 ± 2.06 ^b^	40.40 ± 2.25 ^b^
SA1:9	35.37 ± 0.12 ^a^	11.20 ± 0.10 ^a^	5.89 ± 0.12 ^a^	34.23 ± 0.31 ^a^	66.90 ± 0.26 ^a^

All the values are mean ± standard deviation (*n* = 3). Different letters in the same column indicate significant statistical differences (*p* < 0.05). SA10:0, SA9:1, SA7:3, SA5:5, SA3:7, and SA1:9 represent microcapsules with the weight ratio of sodium caseinate to acetylated mung bean starch of 10:0, 9:1, 7:3, 5:5, 3:7, and 1:9, respectively.

## Data Availability

The data presented in this study are available on request from the corresponding author.
